# Causal effects of glycemic traits and endometriosis: a bidirectional and multivariate mendelian randomization study

**DOI:** 10.1186/s13098-024-01311-1

**Published:** 2024-03-27

**Authors:** Qing Xin, Hao-Jia Li, Hao-Kai Chen, Xiao-Feng Zhu, Lin Yu

**Affiliations:** 1 Department of Obstetrics, Guangdong Provincial Key Laboratory of Major Obstetric Diseases, Guangdong Provincial Clinical Research Center for Obstetrics and Gynecology, Guangdong-Hong Kong-Macao Greater Bay Area Higher Education Joint Laboratory of Maternal-Fetal Medicine, The Third Affiliated Hospital of Guangzhou Medical University, Guangzhou, China; 2 Department of Obstetrics and Gynecology, Guangdong Provincial Key Laboratory of Major Obstetric Diseases, Guangdong Provincial Clinical Research Center for Obstetrics and Gynecology, Guangdong-Hong Kong-Macao Greater Bay Area Higher Education Joint Laboratory of Maternal-Fetal Medicine, The Third Affiliated Hospital of Guangzhou Medical University, Guangzhou, China; 3 Department of Clinical Medicine, the Third Clinical School of Guangzhou Medical University, Guangzhou, China; 4 Department of Clinical Medicine, The Nanshan College of Guangzhou Medical University, Guangzhou, China

**Keywords:** Endometriosis, Glycemic traits, Mendelian randomization, Diabetes

## Abstract

**Background:**

Observational studies have suggested an association between endometriosis and glycemic traits, but causality remains unclear. We used bidirectional and multivariate Mendelian randomization (MR) to examine the causal effect of glycemic traits on endometriosis and vice versa.

**Methods:**

We obtained genome-wide association studies summary data of endometriosis and glycemic traits in our study. Inverse variance weighted (IVW), Weighted median, MR-Egger and Mendelian randomization pleiotropy residual sum and outlier (MR-PRESSO) were applied in bidirectional two-sample MR analyses. MVMR was implemented to estimate the causal effect for fasting insulin (FI), fasting glucose (FG), and glycosylated hemoglobin A1c (HbA1c) on endometriosis. To test the validity of our findings, a number of sensitivity analyses were conducted.

**Results:**

The risk of endometriosis was significantly increased by genetically predicted T1DM (OR = 1.02, 95% CI 1.00-1.04, *p* = 0.0171, q = 0.0556) and GDM (OR = 1.01, 95% CI 1.01–1.02, *p* = 1.34 × 10^− 8^, q = 1.74 × 10^− 7^). Endometriosis had a suggestive association with HbA1c (Beta = 0.04, 95% CI 0.00-0.08, *p* = 0.0481, q = 0.1251). Using multivariate Mendelian randomization (MVMR), a significant causal effect of FI on genetically predicted endometriosis was found (OR = 2.18, 95% CI 1.16–4.09, *p* = 0.0154, q = 0.0547). Moreover, no causal associations between endometriosis and other glycemic traits were detected.

**Conclusion:**

Our findings supported the significant causal associations of T1DM, GDM and FI with endometriosis, respectively. Additionally, a suggestive association was found of endometriosis on HbA1c. Importantly, our study may shed light on etiology studies and clinical management of endometriosis.

**Supplementary Information:**

The online version contains supplementary material available at 10.1186/s13098-024-01311-1.

## Introduction

Endometriosis is a chronic, estrogen-dependent inflammatory disease characterized by endometrial-like tissue (stroma and glands) located outside the uterus [1]. Approximately 10% of reproductive aged women suffer from endometriosis with an additional inclination to exhibit infertility, fatigue, multisite pain, and other conditions [2]. The etiology of endometriosis remains unclear. However, increasing evidence indicates that it is a multifactorial disease, with genetic, environmental, immunologic, and inflammatory factors all contributing to its pathogenesis [3], which may account for certain comorbidities.

Diabetes, including type 1 diabetes mellitus (T1DM), type 2 diabetes mellitus (T2DM), and gestational diabetes mellitus (GDM), is a chronic metabolic disease with hyperglycemia that can develop multiple complications and cause adverse maternal and infant outcomes [4]. With an estimated number of 451 million patients worldwide [5], diabetes poses a considerable challenge to public healthcare.

Currently, associations between endometriosis and glycemic traits remain inconsistent. It was generally recognized that women diagnosed with endometriosis do not have an increased risk of developing T2DM [6]. However, according to a cohort study from Nurses’ Health Study II, endometriosis was related with a modest increased risk of T2DM, among subgroups at lower risk for T2DM [7]. Besides, little is known about the comorbidity with endometriosis and T1DM till now, even though their potential shared pathophysiology has been discussed elsewhere [8]. A meta-analysis in 2018 concluded that no correlation between endometriosis and elevated risk of GDM was discovered [9]. Nonetheless, a newly published meta-analysis study indicated significantly higher risk of GDM in women with endometriosis based on 18 studies involving 4,600,885 individuals, which shifted the viewpoint [10].

Mendelian randomization (MR) has been recognized as a powerful methodology simulating randomized controlled trials to identify causality on basis of genome-wide association studies (GWAS) [11]. Multivariate mendelian randomization (MVMR) is an emergent MR methodology that permits the simultaneous assessment of the contribution of relevant exposures to outcomes by integrating the genetic variation of multiple exposures into the same model to minimize the interference of confounding factors [12]. To clarify these associations, we applied a bidirectional two-sample MR to investigate the causal effects of glycemic characteristics, including T1DM, T2DM, GDM, fasting glucose (FG), fasting insulin (FI), and glycosylated hemoglobin A1c (HbA1c), on endometriosis, and vice versa. Given the potential for confounding factors among some glycemic traits, MVMR was implemented to estimate the causal effect for FI, FG and HbA1c on endometriosis.

## Method

### Study design

**Fig. 1:**
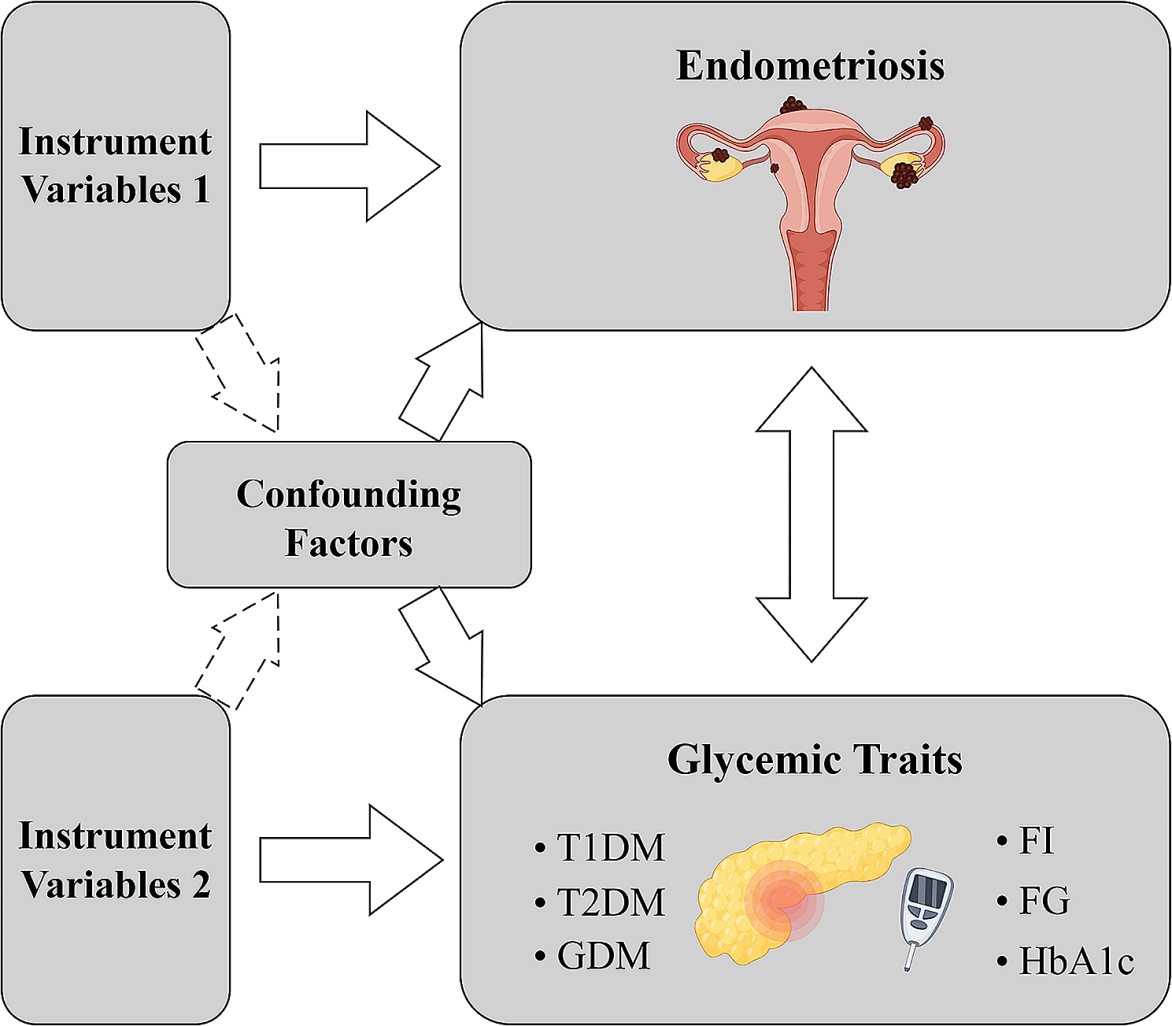
The overall workflow of the study T1DM, Type 1 diabetes mellitus; T2DM, Type 2 diabetes mellitus; GDM, Gestational diabetes mellitus; FI, Fasting insulin; FG, Fasting glucose; HbA1c, Hemoglobin A1c

In the present study, we investigated the causal associations between glycemic traits and endometriosis through bidirectional univariable Mendelian randomization (UVMR). Additionally, MVMR was employed to further evaluate the causal association of FI, FG and HbA1c on endometriosis. To perform MR analysis, three key assumptions need to be met [11]: (1) There is a significant association between exposure and single nucleotide polymorphism (SNP) used as instrumental variables (IVs). (2) IVs are independent of other confounders. (3) The only way IVs affect outcomes is through exposure. Fig. 1 showed the design flowchart of the study.

### Date sources

Traits associated with glycemic traits in this study included: T1DM, T2DM, GDM, FI, FG and HbA1c. Genome-wide association study (GWAS) data for T1DM and FI were obtained from Meta-analyses conducted by Inshaw et al. [13] and Chen et al. [14], respectively. Genetic instruments data for T2DM, GDM, FG and HbA1c were obtained from summary statistics of the DIAGRAM, UK Biobank, MAGIC and Within family GWAS consortium, respectively. In addition, GWAS data for endometriosis were obtained from the FinnGen consortium. The diagnosis of all diseases in this study was based on the International Statistical Classification of Diseases and Related Health Problems. The details of the traits were summarized in Table 1.

**Table 1:** Description of traits used in MR analyses

Traits	Data source	Sample size	Ancestry	PubMed ID
Type 1 diabetes mellitus	Meta-analysis	17,685	European	33830302
Type 2 diabetes mellitus	DIAGRAM	110,452	European	24509480
Gestational diabetes mellitus	UK Biobank	247,540	European	34737426
Fasting insulin	Meta-analysis	151,013	European	34059833
Fasting glucose	MAGIC	133,010	European	22885924
Hemoglobin A1c	Within family GWAS consortium	45,734	European	35534559
Endometriosis	FinnGen	77,257	European	NA
leptin	GWAS Catalog	56,802	European	32917775
adiponectin	ADIPOGen	39,883	European	22479202
resistin	GWAS Catalog	3,301	European	29875488
IL-1B	GWAS Catalog	3,301	European	29875488
IL-6	GWAS Catalog	8,189	European	27989323
IL-8	GWAS Catalog	21,758	European	33067605
IL-10	GWAS Catalog	7,681	European	27989323
TNF-α	GWAS Catalog	3,454	European	27989323

### Selection of instrumental variables

Based on the three fundamental assumptions of the MR analysis, a rigorous selection process was conducted to ensure the reliability of the causal association between glycemic traits and endometriosis. Firstly, a significance threshold of p-value < 5e-8 was applied to select SNPs significantly associated with T1DM, T2DM, FI, FG, and HbA1c. Due to the limited number of SNPs meeting the traditional GWAS significance threshold for GDM and endometriosis, we relaxed the threshold to 5e-5 for GDM and 5e-7 for endometriosis. Subsequently, we used a linkage disequilibrium threshold of r2 = 0.001 and a clumping window of 10,000 kb to clump SNPs. Only SNPs that had a significant association with the outcome (*p* < 5e-8) were kept, and the rest were removed. Furthermore, phenoScaner V2 tool was used to exclude SNPs associated with potential confounders. We used the F-statistic to check how strong the IVs were, so that we could avoid bias in estimating the associations. SNPs with F-statistics greater than 10 were considered strong IVs, while weak IVs (F-statistics < 10) were excluded. To ensure consistency, summary statistics were harmonized, and palindromic SNPs were removed to ensure that each SNP was associated with the same effect allele.

### Univariable Mendelian Randomization

Inverse variance weighted (IVW) was implemented as the main MR method to estimate the association between glycemic traits and endometriosis. The IVW approach incorporates the Wald ratio assessment for each SNP into the meta-analysis, which means that it provides estimates that are not affected by horizontal pleiotropy. In addition, Weighted median, MR-Egger and Mendelian randomization pleiotropy residual sum and outlier (MR-PRESSO) were used as complementary approaches to the IVW. These methods could offer more reliable causal estimates across a wider range of scenarios. The weighted median approach is beneficial to handling potentially invalid instruments, as it can provide reliable causal estimates even when a substantial proportion of the instruments are invalid, up to a threshold of less than 50% [15]. It ensures that the estimation of causal links remains precise despite the presence of such instruments. MR-Egger, on the other hand, permits genetic variants to exhibit pleiotropy under the assumption that pleiotropic effects are independent of variant-exposure associations [16]. This feature enables the utilization of intercept terms to evaluate pleiotropy. In cases where the results from these MR methods were inconsistent, IVW was prioritized as the primary outcome.

### Multivariable Mendelian Randomization

When there may be an association between multiple exposures, the application of MVMR can contribute to address potential horizontal pleiotropy and provide more accurate results. MVMR allows for the inclusion of multiple instrumental variables without considering their associations with the exposures of interest. In this study, considering the association among FI, FG and HbA1c, we employed MVMR to incorporate all instrumental variables for the three exposures to assess their independent effects on endometriosis.

### Sensitivity analyses

To test the validity of our findings, we conducted a number of sensitivity analyses. Firstly, the Cochran’s Q statistic of the IVW method were utilized to identify the heterogeneity of IVs. A p-value of 0.05 or less indicated significant heterogeneity, while a p-value above 0.05 suggested no evidence of heterogeneity [17]. Secondly, we conducted MR Egger intercept test and MR-PRESSO global test to assess the horizontal pleiotropy in the IVs and p-value greater than 0.05 indicated no horizontal pleiotropy detected [18]. In addition, MR-PRESSO analysis was used to identify and remove significant outlier SNPs that may introduce horizontal pleiotropy. Finally, to evaluate the effect of each SNP on the observed associations, we conducted leave-one-out analyses. This approach involves removing one SNP at a time from the analysis and repeating the association test.

### Risk factors analysis

In this study, we investigated the genetic mechanisms underlying the association between endometriosis and diabetes. We selected leptin, adiponectin, resistin, interleukin-1B (IL-1B), interleukin-6 (IL-6), interleukin-8 (IL-8), interleukin-10 (IL-10) and tumor necrosis factor alpha (TNF-α) as potential mediators for analysis. We performed MR analyses on these factors, as well as T1DM and GDM. Table 1 shows the detailed information on each data source. We used IVW estimates as the main results. The significance level was set at *P* < 0.05.

### Statistical analysis

Considering the errors introduced by multiple testing, the false discovery rate (FDR) correction was employed to establish correction thresholds for the results of the MR analyses at each association. We applied the q-value procedure to correct for FDR and used a FDR-corrected threshold of q-value < 0.1 to identify significant associations [19]. We also considered p-value < 0.05 as suggestive evidence of association for glycemic traits and endometriosis, even if the q-value was > 0.1. We performed all statistical analyses using R version 4.2.2, mainly using the following packages: TwosampleMR, MendelianRandomization, MVMR, and MR-PRESSO.

## Result

### Overview

We illustrated the overall workflow of the present study in Fig. 1. Following the SNP screening methodology established previously, a total of 244 SNPs associated with the six glycemic traits phenotypes were selected as IVs. The F-statistics for each SNP were substantial, indicating that the results are unlikely to be influenced by weak instrumental bias. More details on instrumental variables are available in Additional file 1: Table S3-4.

### Causal association between glycemic traits and endometriosis

#### Univariable MR

**Fig. 2:**
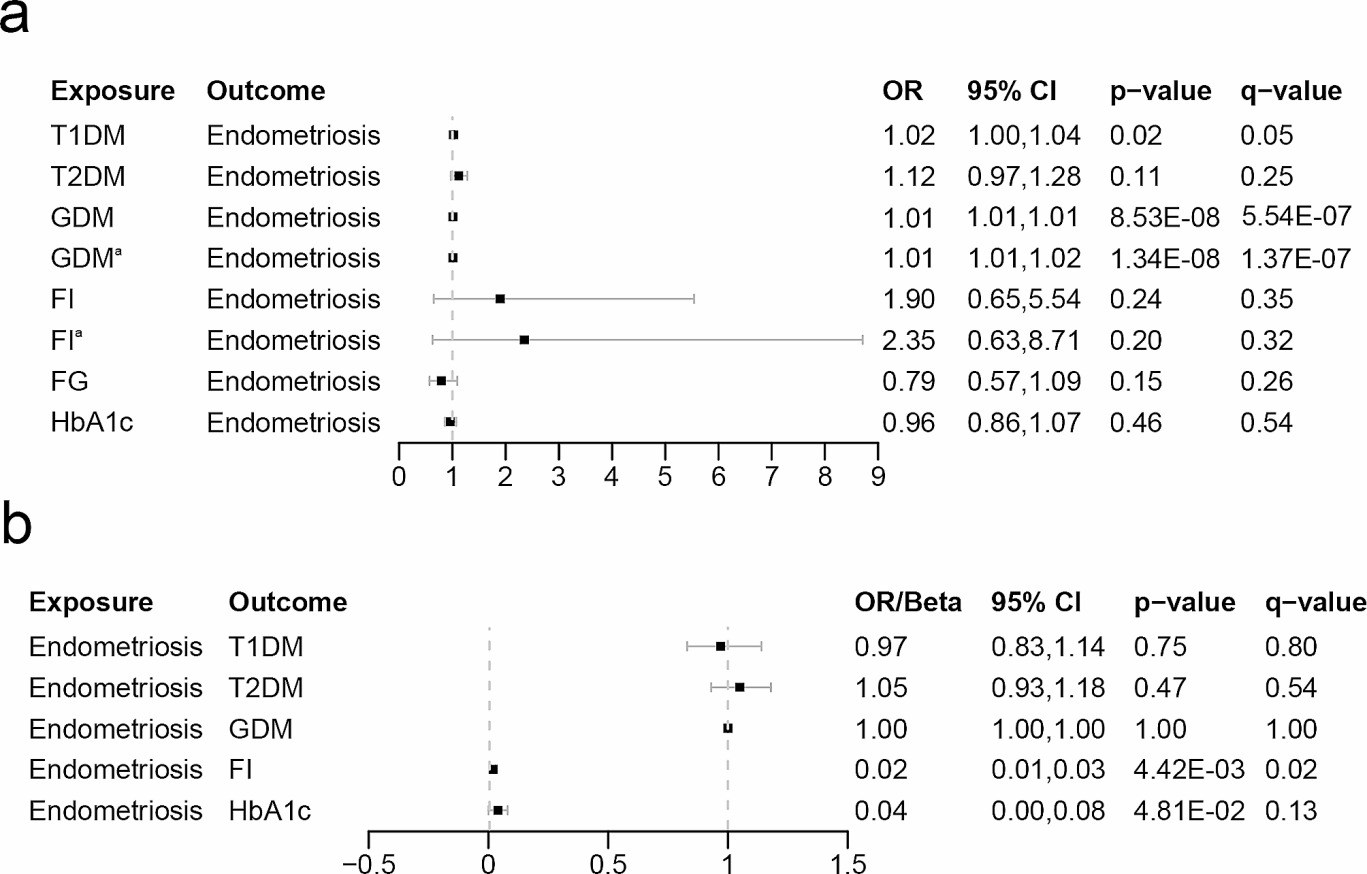
Association of glycemic traits and endometriosis in UVMR analyses ^a^After removing outliers from MR-PRESSO outlier test. The ORs in this study show the impact of per log-OR rise in glycemic traits on endometriosis (**a**) and vice versa (**b**). These ORs were derived from an inverse-variance weighted technique. T1DM, Type 1 diabetes mellitus; T2DM, Type 2 diabetes mellitus; GDM, Gestational diabetes mellitus; FI, Fasting insulin; FG, Fasting glucose; HbA1c, Hemoglobin A1c

**Fig. 3:**
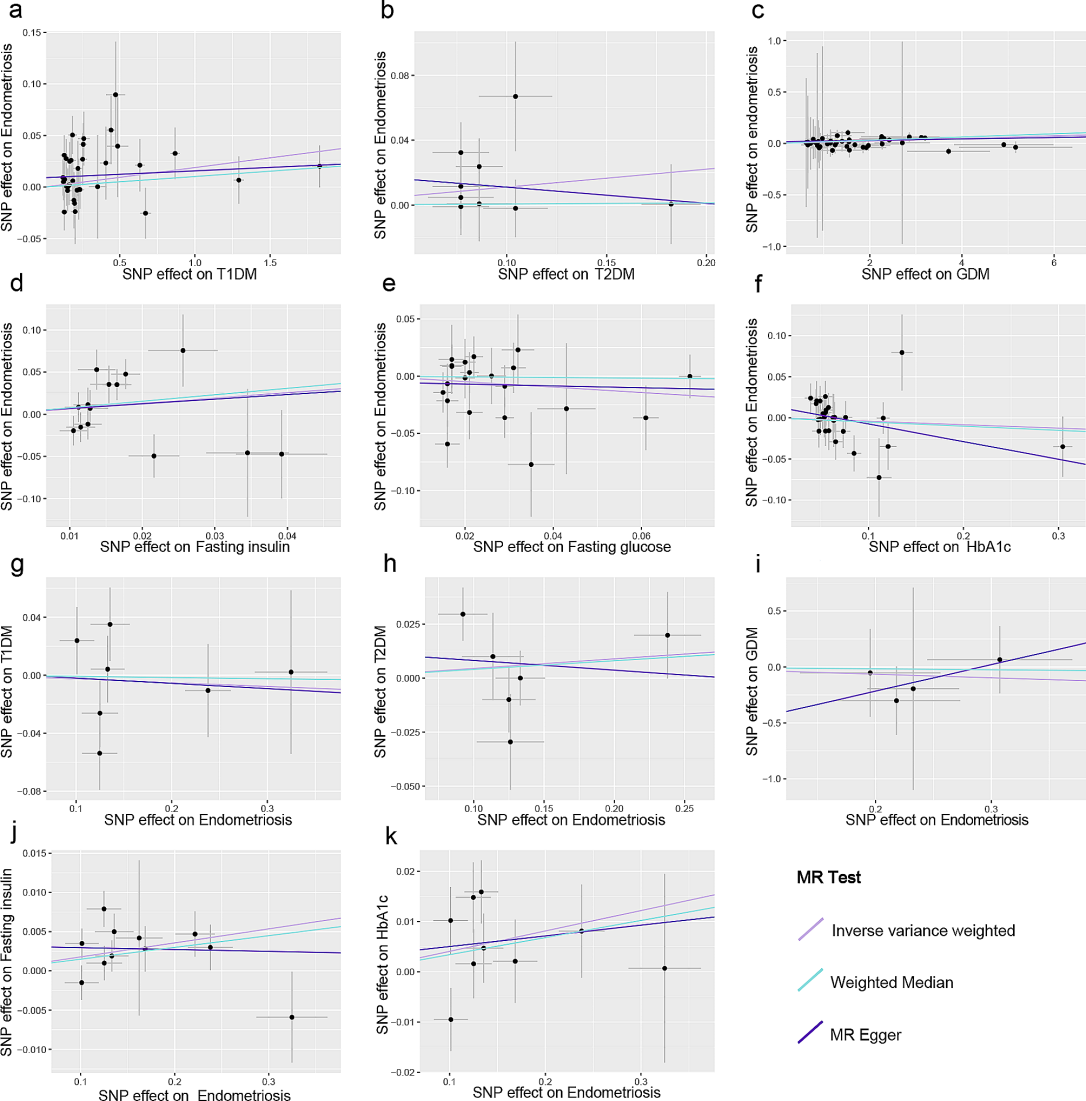
Scatter plots of UVMR (**a**) for T1DM on endometriosis; (**b**) for T2DM on endometriosis; (**c**) for GDM on endometriosis; (**d**) for FI on endometriosis; (**e**) for FG on endometriosis; (**f**) for HbA1c on endometriosis; (**g**) for endometriosis on T1DM; (**h**) for endometriosis on T2DM; (**i**) for endometriosis on GDM; (**j**) for endometriosis on FI; (**k**) for endometriosis on HbA1c. For each SNP, the 95% confidence intervals are shown by the lines that are horizontal and vertical. The correlations’ strength, which was calculated using various methods, is indicated by the solid lines’ slopes. T1DM, Type 1 diabetes mellitus; T2DM, Type 2 diabetes mellitus; GDM, Gestational diabetes mellitus; FI, Fasting insulin; FG, Fasting glucose; HbA1c, Hemoglobin A1c

The association between glycemic traits and endometriosis estimated by UVMR is shown in Figs. 2 and 3 and Additional file 1: Table S1. The risk of endometriosis was significantly increased by genetically predicted T1DM (OR = 1.02, 95% CI 1.00-1.04, *p* = 0.0171, q = 0.0556) and GDM (OR = 1.01, 95% CI 1.01–1.02, *p* = 8.53 × 10^− 8^, q = 5.54 × 10^− 7^), according to the IVW estimates. After removing the outlier SNPs (rs146550543, rs146944614, rs146952957, rs187721033 rs537531044, rs74849261) using MR-PRESSO, the association between GDM and endometriosis remained stable (OR = 1.01, 95% CI 1.01–1.02, *p* = 1.34 × 10^− 8^, q = 1.74 × 10^− 7^). The results from other MR methods presented consistent directions with the causal estimate of IVW, further supporting the robustness of the IVW results (Additional file 1: Table S1). The causal effect of endometriosis on FI was significant in the reverse MR analysis, as indicated by the IVW estimate (Beta = 0.02, 95% CI 0.01–0.03, *p* = 0.0044, q = 0.0191). However, there was inconsistency in the beta direction between MR Egger and other MR models for the association between endometriosis and FI (Additional file 1: Table S1). In addition, endometriosis was considered to have a suggestive association with HbA1c after FDR correction (Beta = 0.04, 95% CI 0.00-0.08, *p* = 0.0481, q = 0.1251). Notably, the reverse causal association between endometriosis and FG was not assessed due to insufficient SNPs. Moreover, causal associations between endometriosis and other glycemic traits were not found.

#### Multivariable MR

The estimated association between glycemic traits and endometriosis using MVMR was presented in Table 2. A significant causal effect of FI on genetically predicted endometriosis was found in this study (OR = 2.18, 95% CI 1.16–4.09, *p* = 0.0154, q = 0.0547), demonstrating consistent directionality with the propensity for causal estimation observed in UVMR.

**Table 2:** Multivariate mendelian randomization estimates for the causal association between glycemic traits and endometriosis

Exposure	Outcome	nSNP	OR	95% CI	*p*-value	*q*-value	F-statistic
FI	Endometriosis	24	2.18	1.16–4.09	0.0154	0.0547	26.53
FG	Endometriosis	27	0.70	0.44–1.11	0.1278	0.2556	16.56
HbA1c	Endometriosis	18	1.15	0.88–1.50	0.3065	0.4086	11.27

SNP, single nucleotide polymorphism; OR, odd ratio; 95%CI, 95% confidence interval; FI, Fasting insulin; FG, Fasting glucose; HbA1c, Hemoglobin A1c.

#### Sensitivity analyses

Sensitivity analyses were performed for UVMR with the details listed in Table 3. The results of Cochrane’s Q test suggested the presence of heterogeneity among the IVs used to estimate the effect of endometriosis on GDM and FI. However, as random-effects IVW allows for the estimation of causal associations in the presence of heterogeneity, the findings of this study remain reliable. Outliers were identified in the MR-PRESSO global test, with 6 outliers (rs146550543, rs146944614, rs146952957, rs187721033, rs537531044, rs74849261) detected for the association between GDM and endometriosis, and 2 outliers (rs1474696, rs860598) detected for the association between FI and endometriosis. The association between GDM and endometriosis showed evidence of pleiotropy, but the MR-Egger intercept tests did not indicate any significant pleiotropic effects (p-value > 0.05). This suggests that the causal relationship between GDM and endometriosis is not confounded by pleiotropy. After the exclusion of outlier from MR-PRESSO global test, no significant pleiotropic effects were observed between GDM and endometriosis. No single SNP was found to drive the causal associations in the study, as suggested by the leave-one-out method.

**Table 3:** Heterogeneity, pleiotropy, and MR-PRESSO global tests for the univariable mendelian randomization

Exposure	Outcome	Heterogeneity test		Pleiotropy test		MRPRESSO	
		Q value	*p*-value	Intercept	*p*-value	oulier	*p*-value
T1DM	Endometriosis	33.68	0.43	0.01	0.07	0	0.29
T2DM	Endometriosis	6.70	0.57	0.02	0.40	0	0.86
GDM	Endometriosis	1502.68	0.00	-0.20	0.00	6	0.01
GDM^a^	Endometriosis	56.18	0.04	0.02	0.46	0	0.05
FI	Endometriosis	55.24	0.00	0.01	0.72	2	< 2E-04
FI^a^	Endometriosis	26.52	0.01	0.00	0.96	0	0.02
FG	Endometriosis	21.38	0.38	-0.01	0.61	0	0.50
HbA1c	Endometriosis	22.84	0.64	0.01	0.12	0	0.72
Endometriosis	T1DM	8.74	0.19	0.00	0.97	0	0.26
Endometriosis	T2DM	8.31	0.14	0.01	0.70	0	0.21
Endometriosis	GDM	0.88	0.83	-0.69	0.55	0	0.75
Endometriosis	FI	15.10	0.13	0.00	0.30	0	0.17
Endometriosis	HbA1c	11.27	0.19	0.00	0.79	0	0.72

^a^After removing outliers from MR-PRESSO outlier test. T1DM, Type 1 diabetes mellitus; T2DM, Type 2 diabetes mellitus; GDM, Gestational diabetes mellitus; FI, Fasting insulin; FG, Fasting glucose; HbA1c, Hemoglobin A1c; MR-PRESSO, Mendelian randomization pleiotropy residual sum and outlier; Q value, the statistics of Cochran’s Q test.

Additional file 1: Table S2 presents the results of the MVMR sensitivity analysis. Cochrane’s Q test indicated the presence of heterogeneity among the instrumental variables (IVs). The MR-Egger intercept test did not reveal any significant pleiotropic effects, suggesting that the observed associations are not due to pleiotropy.

#### Risk factors analysis

We aimed to elucidate the genetic mechanisms underlying the association of endometriosis with T1DM and GDM. For this analysis, we chose leptin, adiponectin, resistin, IL-1B, IL-6, IL-8, L-10 and TNF-α as potential mediators. However, none of these mediators showed significant evidence of association between T1DM or GDM and endometriosis. Additional file 1: Table S6 provides the details of this analysis.

## Discussion

The aim of this study was to examine the causal relationships between endometriosis and glycemic traits using MR approach. Significantly positive causal associations for T1DM, GDM on endometriosis were obtained, respectively. Using MVMR, we found a significant causal effect of genetically predicted fasting insulin (FI) levels on the risk of endometriosis. Additionally, suggestive association was exhibited for endometriosis on HbA1c.

Endometriosis is a gynecologic disease that can seriously affect quality of life for patients. It affects approximately 5%-10% of women in the world [20]. Previous studies have demonstrated that insulin resistance-related disorders may be instrumental in endometriosis. A cohort study conducted in China found that women with endometriosis had higher levels of insulin and plasma glucose [21]. Endometriosis and T1DM have comparable pathophysiologic mechanisms, as both are involved in chronic inflammation induced by immune dysfunction [8, 22]. Another population-based study from Taiwan demonstrated that T1DM can have severe effects on the female reproductive system [23]. The findings above were consistent with the MR evidence. Furthermore, a cohort study on women of reproductive age found that endometriosis increased the risk of GDM, independent of other factors [24]. A meta-analysis also supported a positive relationship between GDM and endometriosis [10]. In our own study, we reported that genetic predisposition to GDM was related to an elevated risk of endometriosis. The underlying pathophysiological mechanism shared by GDM and endometriosis may involve the insufficiency of Vitamin D-binding protein (VDBP) [25, 26]. The evidence collectively indicates a bidirectional causal association between GDM and endometriosis. However, a significant causal effect of endometriosis on GDM was not found in this study using various MR methods. Further research is needed to fully understand the bidirectional causal association between these two conditions. In addition, A case report showed an increased risk for endometriosis on HbA1c levels after receiving a gonadotropin-releasing hormone (GnRH) analog treatment [27]. In our study, genetically predicted endometriosis was suggested associated with an increased risk of HbA1c. However, this causal effect may be masked or exaggerated by the effect of sex hormone therapy although we removed SNPs significantly associated with sex hormones in our study.

Some potential mechanisms may explain the findings of the study. T1DM is considered an autoimmune disease [28]. Toll-like receptors (TLRs) are important protein substances known for their effective role in the human immune system [29]. Researchers have shown that the overexpression of TLR4 may contribute to the progression of T1DM [30]. Additionally, a previous study demonstrated that women with GDM have significantly higher levels of TLR4 and TNF-α compared to healthy individuals [31]. Interestingly, a study from Japan revealed that TLR4 mediated the oxidative stress and inflammatory burden in endometriosis by analyzing the endometrial tissues [32]. This suggests that TLR4 may be a significant factor in the association between endometriosis and diabetes. Furthermore, the gut microbiota is a key factor in controlling the inflammation and protecting the host from chronic disease [33]. Notably, researchers have found that gut microorganisms are altered and dysfunctional in women with endometriosis, similar to diabetic patients [34]. This implies that gut microbiota may be a potential factor in the causal association between endometriosis, diabetes, and their clinical indicators. Estradiol, a hormone, can exacerbate the progression of endometriosis [2]. Previous study has demonstrated that the estrogen receptor was high expressed in the endometriotic tissues, leading to a higher level of inflammation in endometrium [35]. Estrogen signaling can also cause enhance the cell proliferation in the tissues [36]. In addition, an experimental study using porcine coronary arteries revealed that estradiol could influence the activity of the vascular endothelium and promote the oxidative stress. In T1DM patients, estradiol levels are higher compared to the control group [37]. Moreover, based on the vivo experimental model, researchers from Korea found that the placenta and uterus were rebuilt in the rats with GDM, resulting in the upregulation of genes related to estradiol production [38]. This suggests that the estradiol may be a potential mediator between endometriosis and diabetes.

In regard to the MR methods employed in the present study, the IVW method generally has a higher degree of credibility compared to others. The MR-Egger method has wider confidence intervals, making it statistically less efficient [16]. In conclusion, the IVW method is commonly regarded as an effective approach for screening the causal genetics association between variables. A potential limitation of the IVW method is the presence of horizontal pleiotropy, which occurs when the genetic variants used as instrumental variables affect the outcome through pathways other than the exposure of interest. This can bias the causal estimate and lead to erroneous interpretation of the results. Therefore, it is recommended to consider MR-Egger as an additional approach to assess results, as it allows for the detection of horizontal pleiotropic effects that may be unbalanced or directed for all SNPs [39]. Previous studies have highlighted the importance for considering the consistent beta direction in all MR methods [40, 41]. Therefore, when combining the beta value of four MR methods, we did not have sufficient statistical confidence to establish a causal association between endometriosis and FI.

To our knowledge, this study presents the first investigation into the causal association between glycemic traits and endometriosis using MR approaches. Endometriosis is a gynecologic disease with an undetermined etiology, and understanding its underlying causes can greatly contribute to its prevention and treatment in clinical practice. Importantly, our study aimed to provide valuable insights into the etiology of endometriosis and its clinical management. Exploring the molecular biological mechanisms that contribute to these causal associations can effectively assist clinicians in managing patients with comorbidities. The study possesses several strengths. Firstly, the analyses were conducted using GWAS data with large sample size, ensuring the robustness of statistical results. Secondly, a comprehensive range of glycemic traits was included in the analysis, ensuring the breadth of the conclusions. Thirdly, four MR approaches were employed in bidirectional MR analyses, enhancing the credibility of the findings. Lastly, MVMR was implemented to estimate the independent effects of the exposures on endometriosis in this study. However, it is important to acknowledge some limitations. First limitation of our study is the lack of ethnic diversity among the study participants, who were mainly of European ancestry. This may limit the generalizability of our findings to other populations with different genetic backgrounds and environmental exposures. Secondly, the detailed information regarding the specific characteristics and severity of the diseases, such as endometriosis, was unavailable, restricting the analysis to a macro level. Thirdly, due to the limited number of SNPs meeting the traditional GWAS significance threshold of 5e-8, we relaxed the threshold to 5e-5. Despite rigorous multiple test corrections and sensitivity analyses, some degree of bias may still be present.

Our study has implications for the clinical management and care of patients with endometriosis or diabetes. The present MR study has revealed genetically predicted causal associations between glycemic traits, diabetes mellitus, and endometriosis, indicating T1DM and GDM may be risk factors for endometriosis. Clinicians should be alert to potential co-morbidity of T1DM or GDM and endometriosis when patients with high glucose level report symptoms such as dysmenorrhea, pain, or bleeding that is not attributable to other causes. To date, controlling blood glucose level and maintaining a diet with low glycemic index have not been recommended in guidelines on endometriosis management. However, regular screening for diabetes-related indicators may benefit the clinical management of patients with endometriosis, particularly when they have risk factors for diabetes, such as obesity, unhealthy diet, smoking. Further research is warranted to elucidate the association and thus inform the development of optimal management strategies.

## Conclusion

The results of the MR study indicated that T1DM, GDM and FI were causally associated with endometriosis. In addition, endometriosis was suggested associated with FI. MVMR indicated that higher FI levels may increase the susceptibility to endometriosis. Importantly, our study may shed light on etiology studies and clinical management of endometriosis. However, further research is needed to explore potential pathophysiologic mechanisms underlying the relationship between glycemic traits and endometriosis.

### Electronic supplementary material

Below is the link to the electronic supplementary material.

Supplementary Material 1

Supplementary Material 2

## Data Availability

The summary statistics for T1DM, T2DM, GDM, FI and HbA1c are available at https://www.ebi.ac.uk/gwas/home and data for FG is available at https://gwas.mrcieu.ac.uk. The summary statistics for endometriosis is available in FinnGen consortium (https://www.finngen.fi/en/access_results).

## Abbreviations

FG: Fasting glucose
FI: Fasting insulin
HbA1c: Hemoglobin A1c
GDM: Gestational diabetes mellitus
T1DM: Type 1 diabetes mellitus
T2DM: Type 2 diabetes mellitus
