# Supplementary material for: Causal effects of glycemic traits and endometriosis: a bidirectional and multivariate mendelian randomization study
Source: Diabetol Metab Syndr. 2024 Mar 27;16:77. doi: 10.1186/s13098-024-01311-1 (PMC10967113; doi:10.1186/s13098-024-01311-1)
Supplement: Supplementary file 1 — Supplementary Material 1 [file 13098_2024_1311_MOESM1_ESM.docx]

**
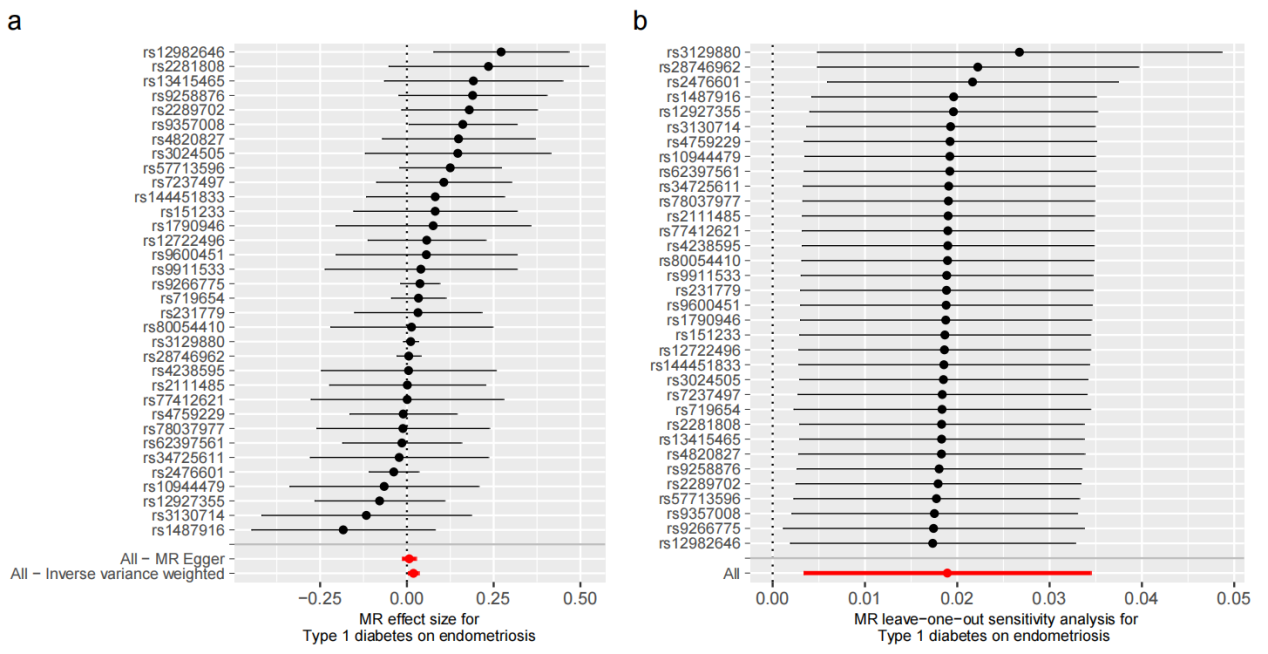
**

**Figure S1.** Forest plot (a) and leave-one-out analysis (b) for Type 1 diabetes on endometriosis.


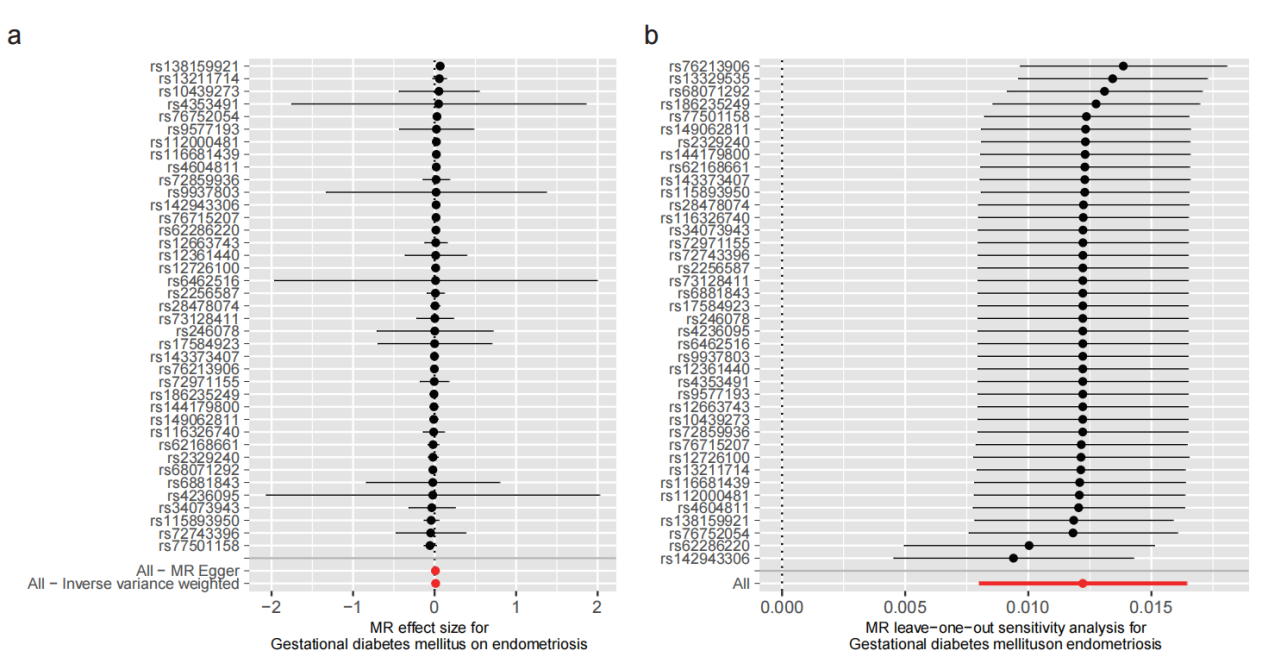


**Figure S2.** Forest plot (a) and leave-one-out analysis (b) for Gestational diabetes mellitus on endometriosis.

**
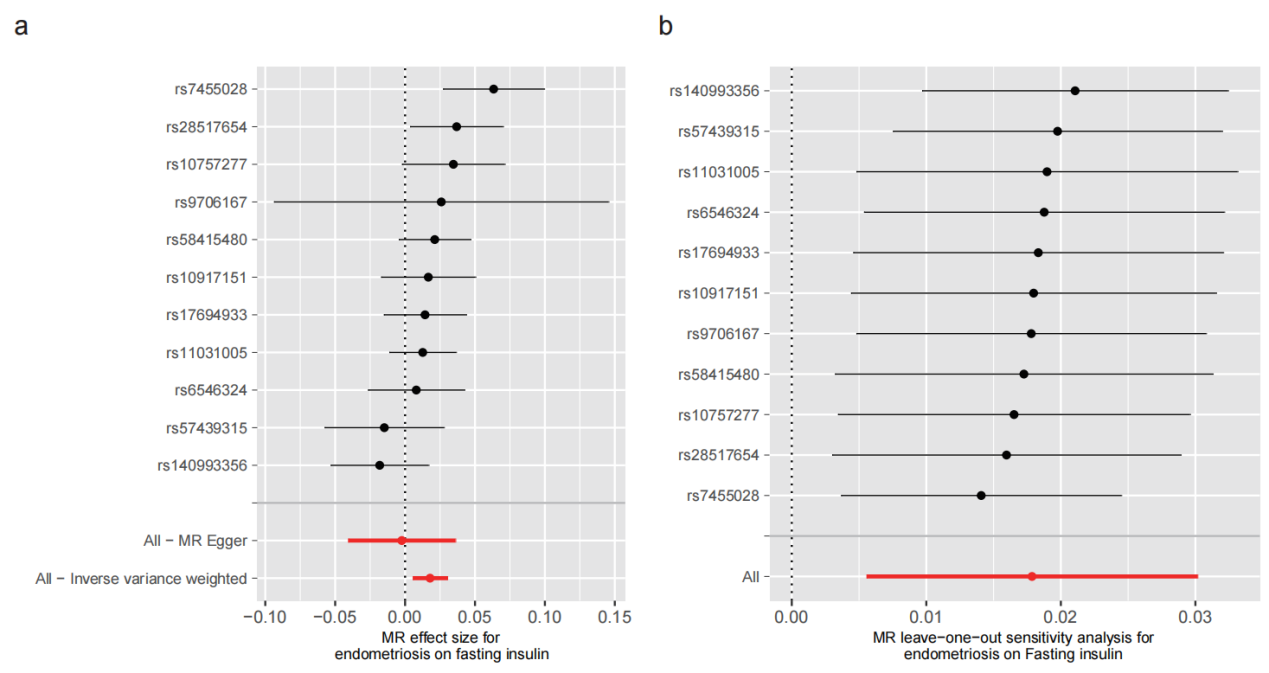
**

**Figure S3.** Forest plot (a) and leave-one-out analysis (b) for endometriosis on fasting insulin.

**
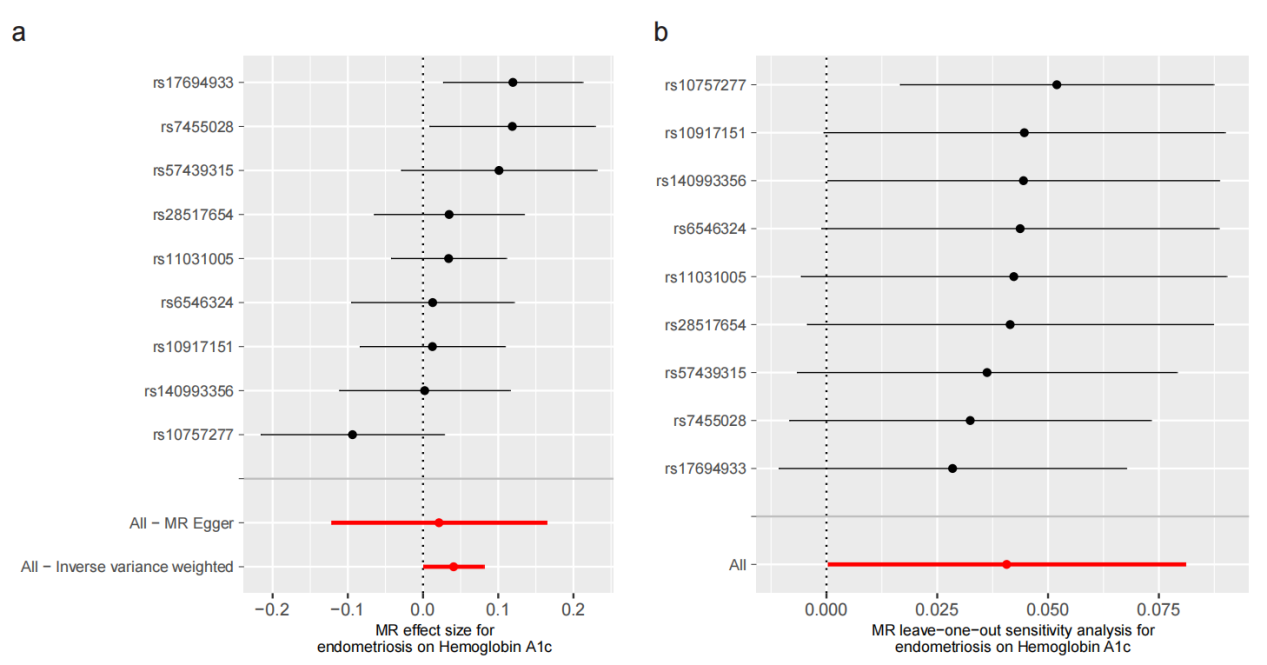
**

**Figure S4.** Forest plot (a) and leave-one-out analysis (b) for endometriosis on Hemoglobin A1c.
